# Association of Intravenous Acetaminophen Administration With the Duration of Intravenous Opioid Use Among Hospitalized Pediatric Patients

**DOI:** 10.1001/jamanetworkopen.2021.38420

**Published:** 2021-12-21

**Authors:** Anita K. Patel, Jiaxiang Gai, Eduardo Trujillo-Rivera, Farhana Faruqe, Dongkyu Kim, James E. Bost, Murray M. Pollack

**Affiliations:** 1 Division of Critical Care Medicine, Department of Pediatrics, Children’s National Health System and George Washington University School of Medicine and Health Sciences, Washington, DC; 2 Children’s National Health System and George Washington University School of Medicine and Health Sciences, Washington, DC; 3 George Washington University School of Medicine and Health Sciences, Washington, DC; 4 Children’s National Health System, Washington, DC; 5 Department of Pediatrics, Children’s National Health System and George Washington University School of Medicine and Health Sciences, Washington, DC

## Abstract

**Importance:**

Adoption of multimodal pain regimens that incorporate nonopioid analgesic medications to reduce inpatient opioid administration can prevent serious opioid-related adverse effects in children, including tolerance, withdrawal, delirium, and respiratory depression. Intravenous (IV) acetaminophen is in widespread pediatric use; however, its effectiveness as an opioid-sparing agent has not been evaluated in general pediatric inpatients.

**Objective:**

To determine if IV acetaminophen administered prior to IV opioids is associated with a reduction in the total duration of IV opioids administered compared with IV opioids administered without IV acetaminophen in general pediatric inpatients.

**Design, Setting, and Participants:**

This comparative effectiveness research study included data on pediatric inpatients from 274 US hospitals between January 2011 and June 2016 collected from a national database. Outcomes were compared with a propensity score–matched analysis of pediatric inpatients administered IV opioids without IV acetaminophen (control) and those administered IV acetaminophen prior to IV opioids (intervention). Data were analyzed from January 2020 through October 2021.

**Exposures:**

Patients in the intervention group received IV acetaminophen prior to IV opioids. Patients in the control group received IV opioids without IV acetaminophen.

**Main Outcomes and Measures:**

Total duration of all IV opioids administered during a patient’s hospitalization.

**Results:**

Of 893 293 pediatric inpatients, a total of 104 579 were included in analysis (median [IQR] age, 1.3 [0-14.7] years; 59 806 [57.2%] female; 21 485 [21.5%] African American, 56 309 [53.8%] White), of whom 18 197 (2.0%) received IV acetaminophen, and 287 504 (34.0%) received IV opioids. After applying exclusion criteria, among patients who received IV acetaminophen, 1739 (10.8%) received IV acetaminophen prior to IV opioids within a median (IQR) treatment time of 1.5 (0.02-7.3) hours. After propensity score matching produced comparable groups in the control and intervention groups (with 839 patients in each group), the multivariable model estimated a 15.5% shorter duration of IV opioid use in the intervention group, with an absolute IV opioid reduction of 7.5 hours (95% CI, 0.7-15.8 hours).

**Conclusions and Relevance:**

In this comparative effectiveness study, IV acetaminophen administered prior to IV opioids was associated with a reduction in IV opioid duration by 15.5%. Multimodal pain regimens that use IV acetaminophen prior to IV opioids could reduce IV opioid duration.

## Key Points

**Question:**

Does intravenous (IV) acetaminophen administered prior to IV opioids reduce the total duration of IV opioids administered during a patient’s inpatient hospitalization?

**Findings:**

In this comparative effectiveness research study including 893 293 hospitalized children, administration of IV acetaminophen prior to IV opioids was associated with a significant 15.5% reduction in total IV opioid duration when compared with administration of IV opioids alone.

**Meaning:**

The results of this study suggest that IV acetaminophen administered prior to IV opioids should be considered in multimodal pain regimens because it may potentially reduce the total duration of IV opioids administered to hospitalized children.

## Teaser

This comparative effectiveness research study evaluates whether receipt of intravenous acetaminophen before receipt of intravenous opioid is associated with a reduction in intravenous opioid duration.

## Introduction

Opioid medications are a common therapeutic approach to alleviate pain in pediatric inpatients. A recent assessment of medication use in pediatric intensive care unit (ICU) patients found that 39.4% of all patients received opioid medications.^1^ While effective, opioid use may lead to adverse consequences including dependence, tolerance, and withdrawal, which can prolong ICU and hospital length of stay.^2,3,4,5,6^ Important short-term adverse effects include delirium, respiratory depression, oversedation, delayed gut motility, and urinary retention.^6^ Children experience these complications more frequently than adults, possibly because of developmental differences in metabolism, excretion, receptor subtypes, signal transduction, receptor induction, and cellular regulatory pathways.^7,8,9^

The importance and frequency of clinical opioid adverse effects and increased hospital length of stay has led to attempts to reduce opioid use. A prominent therapeutic approach uses multiple medications, most notably a nonopioid analgesic medication (with opioids reserved as second-line analgesic medications).^10,11,12^ Intravenous (IV) acetaminophen has been a common analgesic initiated prior to opioids in multimodal pain regimens with the purpose of reducing subsequent opioid requirements.^13,14,15,16^ However, assessments of the association between IV acetaminophen and opioid use in multimodal pain regimens have had conflicting results.^13,14,15,17,18,19,20,21^ In particular, randomized studies that compare treatment that initiates pain control with IV acetaminophen and supplements with opioids vs therapy initiated with opioids have been small with conflicting results.^13,18,19,22^ The aim of this analysis was to determine if the initiation of IV acetaminophen prior to IV opioids would result in a reduction in the total hospital duration of IV opioid use compared with IV opioid medications administered without IV acetaminophen in both operative and nonoperative pediatric inpatients in a national database. This analysis used a national database and propensity score matching.

## Methods

For this comparative effectiveness study, we followed the International Society for Pharmacoeconomics and Outcomes Research (ISPOR) reporting guideline for nonrandomized studies using large data sets.^23^ We defined the research question a priori, reported all research methodology, had no changes or modifications to our prespecified plan, and included interpretations to our findings in the discussion to aid in dissemination of this work to patient care and future research studies. The institutional review board at Children’s National Health System approved the study and granted a waiver of consent because data were deidentified.

### Database

The data set was derived from the Health Facts database (Cerner Corporation) that collects comprehensive deidentified clinical data on patient encounters from hospitals in the US with a Cerner data use agreement. Data are episodic and longitudinal and include date and time-stamped data including admission and demographic data, laboratory results, medication data derived from pharmacy records, diagnostic and procedure codes, vital signs, respiratory data, hospital outcome, and hospital and regional characteristics. Cerner Corporation has established Health Insurance Portability and Accountability Act compliance operating policies to establish deidentification of data. The database is representative of the US and inclusive of academic and nonacademic hospitals of varied sizes and locations, making it appropriate for comparative effectiveness research that is generalizable.^24,25,26,27^ The database has been used to study multiple aspects of pediatrics and pediatric medication practices.^1,3,28,29,30,31^ eAppendix 1 in the Supplement provides a detailed description of the data cleaning process, definitions, and hospital characteristics.

### Patient Selection

This assessment compared patients receiving IV opioids without IV acetaminophen (controls) to those receiving IV acetaminophen prior to IV opioids (intervention). The primary inclusion criteria were pediatric inpatients who received IV opioids between January 2011 and June 2016 with complete data on age, race, gender, temperature, *International Classification of Diseases, Ninth Revision* (*ICD-9*) or *International Statistical Classification of Diseases and Related Health Problems, Tenth Revision *(*ICD-10*) diagnostic codes, and hospital and regional characteristics (Figure). Patients with incomplete data were excluded. Patients who received IV acetaminophen after IV opioids were also excluded because the treatment arm required IV acetaminophen to be administered prior to IV opioid medications. Ultimately, 274 hospitals donated data on 104 579 pediatric patients with complete data sets. January 2011 corresponded to the time IV acetaminophen became commercially available. Pediatric patients were defined as younger than 22 years at time of admission, consistent with the American Academy of Pediatrics’s definition of a pediatric patient.^32^

**Figure.**
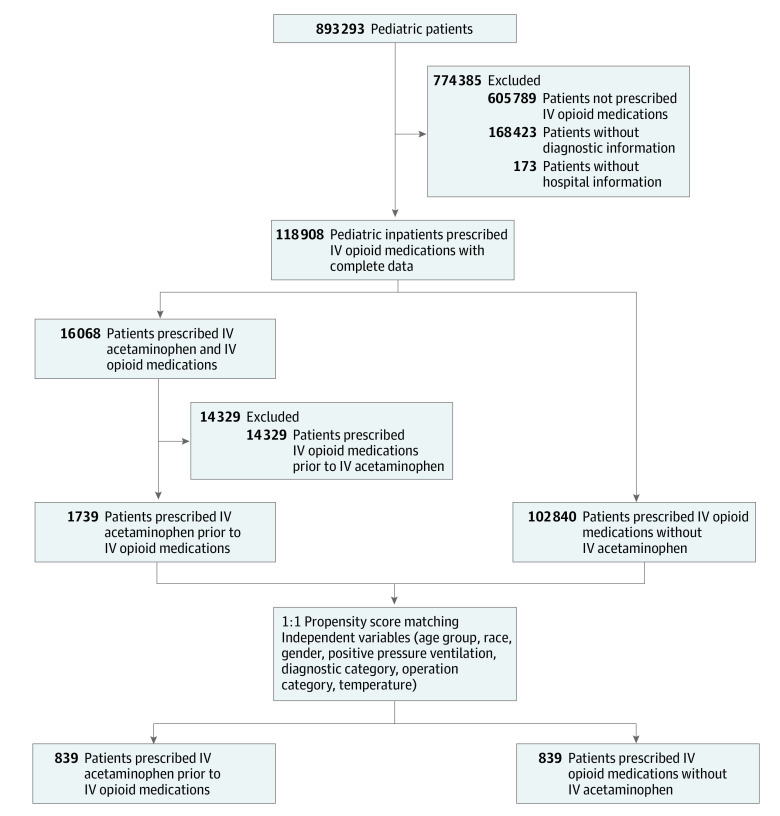
Flowchart of Pediatric Inpatient Inclusion IV indicates intravenous.

### Variables and Outcome Measures

The variables included in the propensity score estimates consisted of age, race, gender, positive pressure ventilation, diagnostic category, operations category, temperature, admission year, hospital census region, hospital bed size, and hospital teaching status (Table 1; eFigure in the Supplement). Race was considered as a variable because previous studies have detailed differing opioid prescribing practices by comparing African American patients with White patients; race was stratified as African American, White, and other (including Asian, Asian and Pacific Islander, biracial, and missing). All variables included in the propensity score estimates were assessed prior to the start of follow-up, which was marked by the first dose of IV opioid in the control group or first dose of IV acetaminophen in the intervention group. Age was categorized into 4 groups: less than 2 years, 2 through 5 years, 6 through 12 years, and 13 up to 22 years. Positive pressure ventilation was identified from the Healthcare Common Procedure Coding System (HCPCS) and *Current Procedural Terminology* (*CPT-4*) codes; invasive and noninvasive positive pressure ventilation were assessed separately. Both the primary diagnosis and operative information were categorized by organ system. The primary diagnoses were categorized into 13 diagnostic groups from the *ICD-9* and *ICD-10* classifications.^33^ When the primary diagnostic group was diseases originating in childbirth or the perinatal period, the secondary and tertiary diagnostic codes were evaluated to classify the primary organ system of dysfunction. Operations were identified from *ICD-9*, *ICD-10-Procedure Coding System*, HCPCS, and *CPT-4* codes. Initial temperature was used as a matching variable because IV acetaminophen is also used for fever. Hospital census region was the greatest level of regional data available in the database, represented by South, Midwest, West, or Northeast. Hospital bed size was categorized into 5 groups: less than 100, 100 to 199, 200 to 299, 300 to 499, and more than 500 beds. Teaching status was characterized as teaching hospital, nonteaching hospital, or unknown.

**Table 1.** Demographic and Clinical Characteristics of Pediatric Inpatients Receiving IV Opioids With and Without IV Acetaminophen

Baseline characteristics	Before propensity score matching			After propensity score matching		
	Control, No. (%) (n = 102 840)^a^	Intervention, No. (%) (n = 1739)^b^	*P* value	Control, No. (%) (n = 839)^a^	Intervention, No. (%) (n = 839)^b^	*P* value
Age, mean (SD), y	6.4 (8.0)	13.4 (6.8)	<.001	13.9 (6.8)	14.0 (6.8)	.20
Age group, y						
<2	16 356 (15.9)	158 (9.1)	<.001	102 (12.2)	83 (9.9)	.28
2 to <6	11 071 (10.8)	190 (10.9)		82 (9.8)	88 (10.5)	
6 to <13	17 392 (16.9)	364 (20.9)		152 (18.8)	175 (20.9)	
13 to <22	58 021 (56.4)	1027 (59.1)		503 (60.0)	493 (58.8)	
Sex						
Female	58 952 (57.3)	854 (49.1)	<.001	388 (46.2)	408 (48.6)	.33
Male	43 888 (42.7)	885 (50.9)		451 (53.8)	431 (51.4)	
Race						
African American	21 485 (21.5)	391 (23.2)	<.001	285 (24.3)	287 (24.5)	.71
White	54 326 (54.3)	1983 (58.3)		695 (59.4)	679 (58.0)	
Other^c^	24 252 (24.2)	313 (18.6)		191 (16.3)	205 (17.5)	
Initial temperature, °C						
<38.0	65 837 (63.6)	977 (56.2)	<.001	0	0	.93
>38.5	1706 (1.7)	54 (3.1)		768 (91.5)	766 (91.3)	
38-38.5	1933 (1.9)	55 (3.2)		39 (4.6)	38 (4.5)	
Missing	33 364 (32.4)	653 (37.6)		32 (3.8)	35 (4.2)	
Mechanical ventilation						
Invasive	4586 (4.5)	39 (2.2)	<.001	19 (2.3)	12 (1.4)	.12
Noninvasive	396 (0.4)	7 (0.4)		1 (0.1)	5 (0.6)	
No ventilation	97 858 (95.2)	1693 (97.4)		819 (97.6)	822 (98.0)	
Diagnoses by system						
Musculoskeletal	4316 (4.2)	130 (7.5)	<.001	41 (4.9)	52 (6.2)	.24
Congenital	16 809 (16.3)	250 (14.4)	.03	145 (17.3)	126 (15.0)	.21
Hematology/oncology	6929 (6.7)	132 (7.6)	.16	61 (7.3)	59 (7.0)	.85
Circulatory	2540 (2.5)	39 (2.2)	.55	19 (2.3)	18 (2.1)	.87
Gastrointestinal	16 334 (15.9)	344 (19.8)	<.001	164 (19.5)	159 (19.0)	.76
Genitourinary	3884 (3.8)	74 (4.3)	.30	38 (4.5)	39 (4.6)	.91
Dermatology	3258 (3.2)	36 (2.1)	<.001	13 (1.5)	18 (2.1)	.37
Nervous	6582 (6.7)	163 (9.4)	<.001	79 (9.4)	85 (10.1)	.62
Endocrine	4250 (4.1)	198 (5.6)	.002	57 (6.8)	47 (5.6)	.31
Injury and poisonings	31 193 (30.3)	341 (19.6)	<.001	161 (19.2)	175 (20.9)	.39
Operations by system						
Endocrine	956 (0.9)	17 (1.0)	.84	10 (1.2)	11 (1.3)	.83
Musculoskeletal	8857 (8.6)	205 (11.8)	<.001	98 (11.7)	91 (10.8)	.59
Cardiovascular	8734 (8.5)	71 (4.1)	<.001	29 (3.5)	29 (3.5)	>.99
Gastrointestinal	9508 (9.2)	160 (9.2)	.95	69 (8.2)	74 (8.8)	.34
Nervous	5198 (5.1)	57 (3.3)	<.001	22 (2.6)	24 (2.9)	.77
Integument	4318 (4.2)	41 (2.4)	<.001	24 (2.9)	17 (2.0)	.27
Genitourinary	3068 (3.0)	50 (2.9)	.79	23 (2.7)	17 (2.0)	.34
Respiratory	3368 (3.3)	40 (2.3)	.02	22 (2.6)	15 (1.8)	.25
Admission year						
2011	17 651 (17.2)	59 (3.4)	<.001	28 (3.3)	31 (3.7)	.18
2012	477 (0.5)	2 (0.1)		0	0	
2013	28 217 (27.4)	381 (21.9)		201 (24.0)	397 (47.3)	
2014	28 217 (27.4)	568 (32.7)		445 (53.0)	2 (0.2)	
2015	20 308 (19.7)	465 (26.7)		1 (0.1)	195 (23.2)	
2016	7907 (7.7)	264 (15.2)		164 (19.5)	214 (25.5)	
Census region						
Midwest	18 911 (18.4)	210 (12.1)	<.001	100 (11.9)	105 (12.5)	.93
North	19 776 (19.2)	625 (35.9)		279 (33.3)	268 (31.9)	
South	31 866 (31.0)	621 (35.7)		319 (38.0)	327 (39.0)	
West	32 287 (31.4)	283 (16.3)		141 (16.8)	139 (16.6)	
Hospital bed size						
<100	24 521 (23.8)	110 (6.3)	<.001	98 (11.7)	105 (12.5)	.98
100-199	8539 (8.3)	182 (10.5)		52 (6.2)	49 (5.8)	
200-299	24 604 (23.9)	283 (16.3)		138 (16.4)	136 (16.2)	
300-499	16 692 (16.2)	960 55.2)		164 (19.5)	167 (19.9)	
≥500	28 484 (27.7)	204 (11.7)		387 (46.1)	382 (45.5)	
Teaching hospital						
Yes	68 515 (66.6)	1489 (85.6)	<.001	714 (85.1)	709 (84.5)	.73
No	33 707 (32.7)	248 (14.3)		125 (14.9)	130 (15.5)	
Unknown	618 (0.6)	2 (0.1)		0	0	
Time interval between IV acetaminophen and first dose of IV opioid, median (IQR), h^d^	NA	1.7 (0.03-8.4)	NA	NA	1.5 (0.02-7.3)	NA
Length of stay, median (IQR), h^d^	70.1 (44.7-129.8)	81.0 (42.0-164.2)	<.001	71.4 (43.9-123.2)	81.6 (42.8-164.1)	.02
Opioid duration, median (IQR), h^d^	30.5 (5.0-74.2)	29.4 (4.0-82.9)	.65	33.5 (5.4-74.0)	27.7 (4.0-76.1)	.41

Abbreviations: IV, intravenous; NA, not applicable.

^a^ Control group includes patients who received IV opioids without IV acetaminophen.

^b^ Intervention group includes patients who received IV acetaminophen followed by IV opioids.

^c^ Other included Asian, Asian and Pacific Islander, biracial, and missing data.

^d^ Time interval between IV acetaminophen and first dose of IV opioid, length of stay, and opioid duration were not included in development of propensity score estimates.

Medication data included the generic and brand name, national drug code, and time the medication was ordered and discontinued. Dosing was not evaluated because reliable per kilogram dosing information was not consistently available. Analgesic medications administered to patients were assessed individually and by medication class by linking the national drug code to the Multum classification (eAppendix 2 in the Supplement).^34,35^ Multum provides information about medications’ therapeutic action in 3 categories, from general mechanism of action to a specific therapeutic category. The duration of medication administration determined from pharmacy records was rounded to the nearest hour. If a single dose of medication was administered, the patient was considered to have received 1 hour of medication. If multiple orders of different IV opioid medications had overlapping administration times, the medication orders were summed and the overall length of administration was determined by the start and end time.

The primary outcome was IV opioid duration, with the first dose of IV opioid administration marking the start of the follow-up period. IV opioid duration was determined by the sum of all IV opioid durations in a patient’s hospitalization. If a patient was simultaneously receiving multiple different generic opioid medications, each medication course was summed to calculate the total duration. Cumulative duration was used as a measure of overall opioid receipt because per kilogram dosing was consistently unavailable.

### Statistical Analysis

A propensity score–matching analysis was performed to ensure balance between the control and intervention groups (eAppendix 2 in the Supplement). Propensity for receipt of IV acetaminophen was modeled using logistic regression for the following variables: age, race, gender, positive pressure ventilation, diagnostic category, operations category, temperature, admission year, census region, bed size, and teaching hospital status using data obtained prior to administration of IV acetaminophen or IV opioid. A 1-to-1 without replacement nearest-neighbor matching method was used. Forest plots were constructed to assess uniformity between the groups before and after matching (eFigure in the Supplement). Categorical variables were expressed as frequency and percentages and were compared using χ^2^ tests (Table 1). Odds ratios with 95% confidence intervals were calculated with a reference group.

Postmatching analyses assessed opioid duration via generalized linear models with log-linked γ distribution. A univariate analysis was first used to assess the association between each variable and opioid duration (Table 2). Those with significant differences were included in the multivariable model comparing the control and intervention groups with regard to IV opioid duration. We fitted a marginal model using hospital system identification numbers as a random effect to account for the clustered data structure. Compound-symmetry structure of covariance matrix was selected (which has constant variance and constant covariance), and empirical sandwich estimator was used to get standard errors of regression coefficients. We used the marginal model (generalized estimating equation type) because the goal was to estimate population-average effect rather than hospital-specific effect. The marginal model produced robust estimates regardless of the choice of covariance structure. *P* value for test of the random effect was <.001, indicating that the clustering effect was needed in the model. Length of stay was the only postbaseline characteristic included in the multivariable model, as it remained significantly different in the propensity-matched sample. Duration was log transformed to ensure normality. All statistical analyses were conducted using SAS software version 9.4 (SAS Institute). Significance was set to *P* < .05 in 2-sided tests.

**Table 2.** Univariate Analysis of Variables Associated With Intravenous Opioid Duration

Covariates	Estimate, mean (SE) [95% CI]	*P* value
Length of stay, h^a^^,^^b^	0.81 (0.03) [0.76 to 0.87]	<.001
Age group, y		
13 to <22	[Reference]	.48
<2	0.08 (0.11) [−0.13 to 0.29]	
2 to 6	0.16 (0.11) [−0.06 to 0.37]	
6 to <13	0.07 (0.09) [−0.10 to 0.24]	
Race		
Other^c^	[Reference]	<.001
African American^d^	−0.195 (0.101) [−0.39 to 0.004]	
White^d^	−0.32 (0.088) [−0.49 to −0.15]	
Initial temperature, °C		
38-38.5	[Reference]	<.001
<38.0^d^	−0.63 (0.17) [−0.95 to −0.30]	
>38.5^b^	0.42 (0.22) [−0.02 to 0.85]	
Mechanical ventilation		
No ventilation	[Reference]	<.001
Invasive^b^	1.53 (0.24) [1.06 to 2.00]	
Noninvasive^d^	−0.54 (0.54) [−1.60 to 0.52]	
Diagnoses by system		
Musculoskeletal	0.15 (0.14) [−0.13 to 0.42]	.30
Congenital^d^	−0.28 (0.09) [−0.45 to −0.10]	.002
Hematology/oncology^b^	0.37 (0.13) [0.12 to 0.62]	.004
Circulatory	−0.10 (0.22) [−0.54 to 0.34]	.65
Digestive	−0.07 (0.08) [−0.24 to 0.09]	.38
Genitourinary	−0.03 (0.16) [−0.34 to 0.28]	.86
Dermatology	−0.31 (0.24) [−0.79 to 0.17]	.20
Nervous^b^	0.37 (0.11) [0.15 to 0.58]	<.001
Endocrine^b^	0.39 (0.136) [0.13 to 0.67]	.004
Injury and poisonings^d^	−0.28 (0.08) [−0.44 to −0.11]	<.001
Operations by system		
Endocrine^d^	0.90 (0.30) [0.33 to 1.48]	.002
Musculoskeletal	0.04 (0.10) [−0.17 to 0.24]	.71
Cardiovascular	1.05 (0.18) [0.70 to 1.40]	<.001
Digestive	0.10 (0.12) [−0.13 to 0.33]	.40
Nervous	0.17 (0.20) [−0.23 to 0.56]	.40
Integument	0.16 (0.21) [−0.26 to 0.58]	.45
Genitourinary	0.04 (0.22) [−0.38 to 0.46]	.86
Respiratory	1.08 (0.22) [0.64 to 1.51]	<.001

^a^ Length of stay was log transformed.

^b^ Variables associated with longer intravenous opioid duration in a univariate analysis included length of stay, temperature greater than 38.5° C, invasive mechanical ventilation, hematology or oncology system diagnoses, nervous system diagnoses, endocrine system diagnoses, endocrine system operations, cardiovascular system operations, and respiratory system operations.

^c^ Other included Asian, Asian and Pacific Islander, biracial, and missing data.

^d^ Variables associated with a shorter intravenous opioid duration in a univariate analysis included African American and White race, temperature less than 38.5° C, noninvasive mechanical ventilation, congenital diagnoses, and injury and poisoning diagnoses.

## Results

### Patient Characteristics

IV opioid duration was evaluated in 104 579 pediatric inpatients from 274 hospitals with a median (IQR) age of 1.3 years (0-14.7 years); a total of 104 579 patients (57.2%) were female, 21 485 (21.5%) were African American and 56 309 (53.8%) were White. The post–propensity score sample included 1678 pediatric inpatients from 53 hospitals with a mean (SD) age of 13.9 (6.8) years, which included 796 (47.4%) female patients (Figure). A total of 893 293 pediatric inpatients were evaluated, of which 287 504 (34.0%) received IV opioids and 18 197 (2.0%) received IV acetaminophen. After excluding patients who did not receive IV opioids, patients without diagnostic information, and patients without hospital information, 118 908 pediatric inpatients received IV opioids, with 16 068 (13.5%) receiving IV acetaminophen. Among patients who received IV acetaminophen, 1739 (10.8%) received IV acetaminophen prior to IV opioids with a median (IQR) of 1.7 hours (0.03-8.4 hours) between IV acetaminophen administration and first dose of IV opioid. The 16 458 patients (89.2%) who received IV acetaminophen after their first dose of IV opioids were excluded from the final population of 104 579 pediatric inpatients who received IV opioids used for further analyses. When compared with those receiving IV opioids only in the univariate model, patients receiving IV acetaminophen followed by IV opioids were older (eg, ages 13 to 22 years: 59.1% [1027 patients] vs 56.4% [58 021 patients]; *P* < .001) and not mechanically ventilated (97.4% [1693 patients] vs 95.2% [97 858 patients]; *P* < .001); and were more likely to have a body temperature above 38° C (3.1% [54 patients] vs 1.7% [1706 patients]; *P* < .001); diagnoses in the musculoskeletal (7.5% [130 patients] vs 4.2% [4316 patients]; *P* < .001), digestive (19.8% [344 patients] vs 15.9% [16 334 patients]; *P* < .001), nervous (9.4% [163 patients] vs 6.7% [6582 patients]; *P* < .001), and endocrine systems (5.6% [198 patients] vs 4.1% [4250 patients]; *P* = .002); and musculoskeletal operations (7.5% [130 patients] vs 4.2% [4316 patients]; *P* < .001) (Table 1).

Propensity score estimates comparing patients administered IV opioid medications without IV acetaminophen (102 840 patients) and those administered IV acetaminophen followed by opioids (1912 patients) showed significant differences in most matching variables (Table 1; eFigure in the Supplement). Propensity score matching produced comparable groups with regard to matching variables, with a sample of 839 patients in each group. After matching, the predominant age group was between ages 13 and 22 years (996 patients [59.4%]), African American individuals represented 24.4% of the populations (572 patients), 796 patients (47.4%) were female, 31 (1.8%) received mechanical ventilator support, and 1534 (91.4%) had temperatures below 38.0° C prior to receipt of IV opioids or IV acetaminophen in the intervention and control groups. The 2 most common diagnostic categories were gastrointestinal disorders (323 patients [19.2%]) and injury and poisonings (336 patients [20.0%]), and the 2 most common operations were musculoskeletal (189 patients [11.3%]) and gastrointestinal (143 patients [8.5%]).

### Opioid Duration

Prior to propensity score matching, the median (IQR) duration of IV opioid use in the control group was 30.5 hours (5.0-74.2 hours) and 29.4 hours (4.0-82.9 hours) in the intervention group (*P* = .65). After propensity score matching, median (IQR) duration of IV opioid use in the control group was 33.5 hours (5.4-74.0 hours) and for the intervention group was 27.7 hours (4.0-76.1 hours) (*P* = .41). The multivariable model after propensity score matching examined differences in IV opioid duration between the control and intervention groups. Significant univariate baseline variables established in a generalized linear mode were included in the multivariable model, which used health system identification numbers as a random effect to account for the clustered data structure (Table 2). We additionally controlled for hospital length of stay that was significantly different before and after propensity score matching (Table 3). After propensity score matching and adjusting for the aforementioned variables, there was a 15.5% shorter duration of IV opioid use in the intervention group vs the control group, an absolute reduction in IV opioid duration by 7.5 hours (95% CI, 0.7-15.8) in the intervention group when compared with the control group.

**Table 3.** Multivariable Analysis of Variables Associated With Intravenous Opioid Duration^a^

Covariate	Estimate (95% CI)^b^	*P* value
Intervention group receiving IV acetaminophen prior to IV opioids	−0.17 (−0.33 to −0.01)	.04
Race		
Other^c^	[Reference]	NA
African American	−0.15 (−0.28 to −0.02)	.02
White	−0.03 (−0.13 to 0.07)	.55
Initial temperature, °C		
38 to 38.5	[Reference]	NA
<38.0	−0.32 (−0.49 to −0.15)	<.001
>38.5	−0.11 (−0.37 to 0.15)	.41
Age group, y		
<2	[Reference]	NA
2 to 6	0.22 (0.03 to 0.40)	.02
6 to <13	0.38 (0.17 to 0.59)	<.001
13 to <22	0.51 (0.33 to 0.69)	<.001
Congenital diagnosis	−0.13 (−0.26 to −0.01)	.04
Length of stay, h^d^	0.89 (0.83 to 0.95)	<.001

Abbreviations: IV, intravenous; NA, not applicable.

^a^ A marginal model was fitted using hospital identification numbers as a random effect to account for the clustered data structure. *P* value for the test of the random effect was <.001, indicating that the clustering effect was needed in the model.

^b^ Confidence intervals derived from Wald tests.

^c^ Other included Asian, Asian and Pacific Islander, biracial, and missing data.

^d^ Length of stay was log transformed.

## Discussion

We assessed the efficacy of IV acetaminophen in reducing overall IV opioid use in pediatric inpatients using a propensity score–matched analysis from a large sample of a national database. To our knowledge, this is the first assessment of the opioid sparing association of IV acetaminophen in a general, real-world pediatric inpatient population. We found that IV acetaminophen administered prior to IV opioids was associated with a 15.5%, or 7.5-hour (95% CI, 0.7-15.8 hours), reduction in total IV opioid duration.

A propensity score–matched analysis is a practical and effective method to reduce selection bias in nonrandomized studies by matching the treatment and control groups for similar propensities for an intervention. In this analysis, they were matched for their propensity to receive IV acetaminophen. The covariates used to estimate the propensity score were age, race, gender, positive pressure ventilation, diagnostic category, operation category, and temperature. The propensity score was then used in a multivariable regression analysis to compare opioid durations when IV acetaminophen was used prior to opioids or not used.

The reduction in IV opioid duration in the group that received IV acetaminophen prior to IV opioid medications confirms several small pediatric and larger adult trials that reported a reduction in opioid utilization when opioids were preceded by IV acetaminophen.^13,14,16,18,20,22,36,37^ Prior pediatric trials of IV acetaminophen primarily focused on specific postoperative patient populations, such as spinal fusions and tonsillectomies, with only 1 trial focusing on nonoperative patients, a cohort of sickle cell patients.^13,14,15,17^ Our inclusion of both operative and nonoperative pediatric inpatients adds to the generalizability of the results. The results of the propensity score method support the general therapeutic approach of incorporating IV acetaminophen prior to IV opioid medications to achieve a reduction in overall opioid use. However, the results of this study may not be applicable to specific subpopulations of pediatric inpatients.

Reducing opioid medication administration can be beneficial in preventing drowsiness, postoperative nausea and vomiting, ileus, respiratory depression, bladder dysfunction, and prolonged immobilization in the short-term as well as long-term dependency.^2,6,38,39^ While opioids have been a mainstay of pain control, recommendations from numerous professional organizations have advocated multimodal pain control regimens such as the one tested in this analysis.^4,40,41^

### Limitations

This study had several limitations, some of which are associated with large data repositories. First, medication use was determined from pharmacy records, not assessed through patient medication administration records. Second, our primary outcome was duration of opioid use, not cumulative opioid dose, because weight-based dosing was not available. Third, the effectiveness of the therapeutic regimens was not assessed because pain scores were unavailable. Fourth, propensity score matching cannot account for unobserved variables, and it is possible that there remain unmeasured differences between the treatment arms that contribute to the observed findings. Fifth, while we believe the results are generalizable, they may not hold for all groups of patients.

## Conclusions

To our knowledge, this is the first study to use a propensity score–matched analysis to evaluate the association of administering IV acetaminophen prior to IV opioid medications with opioid duration in pediatric inpatients. Our results found that IV acetaminophen was associated with an overall reduction in IV opioid duration, suggesting that there could be a benefit to introducing this medication early in multimodal pain regimens, with the ultimate goal of minimizing IV opioid exposure in pediatric inpatients.

## Notes

Supplement. **eAppendix 1.** Overview, Data Cleaning, and Data Definitions for the Health Facts Database **eAppendix 2.** Creation of the Medication Database **eFigure.** Forest Plots of Patients in the Control (IV Opioids Only) and Intervention (IV Acetaminophen Followed by IV Opioids) Groups Pre- and Postpropensity Score Matching
