# Supplementary material for: Association of Intravenous Acetaminophen Administration With the Duration of Intravenous Opioid Use Among Hospitalized Pediatric Patients
Source: JAMA Netw Open. 2021 Dec 21;4(12):e2138420. doi: 10.1001/jamanetworkopen.2021.38420 (PMC8693214; doi:10.1001/jamanetworkopen.2021.38420)
Supplement: Supplement. — eAppendix 1. Overview, Data Cleaning, and Data Definitions for the Health Facts Database eAppendix 2. Creation of the Medication Database eFigure. Forest Plots of Patients in the Control (IV Opioids Only) and Intervention (IV Acetaminophen Followed by IV Opioids) Groups Pre- and Postpropensity Score Matching [file jamanetwopen-e2138420-s001.pdf]

Supplemental Online Content

Patel AK, Gai J, Trujillo-Rivera E, et al. Association of intravenous acetaminophen administration with the duration of intravenous opioid use among hospitalized pediatric patients. *JAMA Netw Open.* 2021;4(12):e2138420. doi:10.1001/jamanetworkopen.2021.38420

eAppendix 1. Overview, Data Cleaning, and Data Definitions for the Health Facts Database

eAppendix 2. Creation of the Medication Database

eFigure. Forest Plots of Patients in the Control (IV Opioids Only) and Intervention (IV Acetaminophen Followed by IV Opioids) Groups Pre- and Postpropensity Score Matching

This supplemental material has been provided by the authors to give readers additional information about their work.

© 2021 Patel AK et al. *JAMA Network Open.*

eAppendix 1. Overview, Data Cleaning and Data Definitions for the Health Facts Database

Health Facts® Data warehouse: The analytic dataset used for this analysis ranged from January 2009 to June 2016. The initial date corresponded to the point when the Health Facts® Data warehouse expanded to include, in general, the current set of data elements. Data cleansing included: a) eliminating duplicate and null values; and b) eliminating data inconsistent with valid entries (e.g. admission times of zero, data inconsistent with life, or negative values). Data cleansing relevant to each data type is detailed in the section for each data type.

Pediatric Patients: < 22 years of age[1]

Inpatient: Inpatients were defined as those having a Health Facts® inpatient classification code or with evidence of care from an Intensive Care Unit (ICU) location including discharge from an ICU care setting, medication requested or dispensed to an ICU care setting, or a laboratory order from an ICU care setting.

Pediatric Intensive Care Unit (ICU) Patients: Patients were identified as the encounters that satisfy all the following characteristics:

- Pediatric patient (above).
- Age in hours not equal to 0.
- ICU patients were identified by the care setting associated with laboratory orders, cares setting to which the medication was requested or dispensed and/or the discharge care setting. The care setting required an ICU designation of at least one of the following: intensive care unit, intensive care unit – cardiac, intensive care unit – medical, intensive

© 2021 Patel AK et al. *JAMA Network Open.*

care unit – neurology, intensive care unit – surgical. Neonatal Intensive Care Unit (NICU) patients were excluded.

- The hospital outcome was known based on the hospital discharge disposition.

Intensive Care Unit Entry and Exit Times: The ICU entry and exit times were determined as follows. The ICU entry time was recorded as the earliest hour based on the laboratory order, medication request or medication dispensed associated with an ICU location (above). Following ICU entry, if there were no laboratory orders, medication requests or medication dispersals, the location was assumed to be the ICU.

We identified the ICU exit time using the following two conditions: First, the medication request, medication dispensed, or laboratory order came from a non-ICU location following ICU care. If the care setting was null or not mapped, the previous care setting was assumed. If multiple care settings including an ICU were observed during the same hour, the care setting was assumed to be an ICU care setting. Second, if the patient was discharged from the hospital from the ICU, the hospital discharge time was used as the ICU discharge time.

Since we recognized that there is imprecision in the assignment of entry and exit times, we assigned a minimum of 10 hours to each ICU stay for survivors (deaths could have a time period < 10 hours). For patients readmitted to the ICU during a hospitalization, a minimum of 40 hours was assigned to each non-ICU time period between ICU admissions. This resulted in 97.0% of PICU patients having only 1 ICU admission, and 2.3% have 2 ICU admissions, and 0.7% have 3 or more. For the rare patient that had more than 5 ICU admissions (0.13%), the ICU admissions after the 5th were not included in the ICU admission.

© 2021 Patel AK et al. *JAMA Network Open.*

Length of ICU stay and time between ICU admissions for those patients with multiple ICU admissions are shown in the table below.

	Median LOS (hours)	IQR (hours)	25%-75% (hours)
First ICU Admission	59	142	21 - 163
Second ICU Admission	38	128	10 - 138
Third ICU Admission	60	175	10 - 185
Time between First and Second ICU Admission	181	297	104 - 401
Time between Second and Third ICU Admission	186	262	118-330

These data are consistent the published literature.[2,3]

Descriptive Data of Hospitals Included in this Report:

All 274 hospitals included in this analysis met inclusion criteria of donating pediatric inpatients with medication data. Descriptive data of these hospitals are included in the table below.

Hospital Characteristics	Number of Patients
Bed Size[1]	
>500	289,050
300 – 499	236,219
200 – 299	176,947
100 – 199	96,511
<100	94,566
Census Region	
South	110
Midwest	66
West	49
Northeast	49
Teaching Hospital	
Yes	650486
No	242807

1. Bed size refers to total number of hospital beds and is not specific to pediatric bed availability. Details about pediatric bed numbers were not available in the Health Facts® database.

Health Facts® Laboratory and Clinical Events (Vital Signs, Respiratory) Data: Laboratory, vital sign, and respiratory data were assessed for measurement units and each test and its specific measurement units were assessed with descriptive statistics and data distributions. Units such as missing or null were combined with the appropriate data when the assessment of the data values, descriptive statistics, and data distributions indicated the identical measurement unit. Laboratory data with several units of measurement were converted to the standard units and combined. This is described in detail in Appendices C and D.

Health Facts® Medication Data: The dataset contains the drug name (generic and brand), national drug code (NDC), medication volume, administration times, and administration frequency that were requested and dispensed from the pharmacy. Medications were assumed to be administered during the times the pharmacy dispensed them to be administered. The duration of medication administration was rounded to the nearest hour. Medications administered once or for less than 1 hour were assigned an administration time of 1 hour. To assess medications by class, the medication table was linked to an industry standard for medication classification, Cerner Multum™ Solutions.[4,5] Cerner Multum™ provides information about therapeutic action in three categories from general mechanism of action to a specific therapeutic category. A total of 96% of medications were classified by Cerner Multum™. The drugs not categorized by Cerner Multum™ were categorized by individual assessment by three physicians (AKP and MMP) into the pre-existing Cerner Multum™ tiered categories. This is described in detail in **Appendix 2.**

© 2021 Patel AK et al. *JAMA Network Open.*

1. Hardin AP, Hackell JM. Age Limit of Pediatrics. *Pediatrics*. 2017;140(3):e20172151. doi:10.1542/peds.2017-2151

2. Edwards JD, Lucas AR, Stone PW, Boscardin WJ, Dudley RA. Frequency, risk factors, and outcomes of early unplanned readmissions to PICUs. *Crit Care Med*. 2013;41(12):2773-2783. doi:10.1097/CCM.0b013e31829eb970

3. Edwards JD, Lucas AR, Boscardin WJ, Dudley RA. Repeated Critical Illness and Unplanned Readmissions Within 1 Year to PICUs. *Crit Care Med*. 2017;45(8):1276-1284. doi:10.1097/CCM.0000000000002439

4. Fung KW, Kapusnik-Uner J, Cunningham J, Higby-Baker S, Bodenreider O. Comparison of three commercial knowledge bases for detection of drug-drug interactions in clinical decision support. *J Am Med Inform Assoc*. 2017;24(4):806-812. doi:10.1093/jamia/ocx010

5. UMLS Metathesaurus - MMSL (Multum) - Synopsis. Accessed July 25, 2018. https://www.nlm.nih.gov/research/umls/sourcereleasedocs/current/MMSL/

© 2021 Patel AK et al. *JAMA Network Open.*

eAppendix 2. Creation of the Medication Database

The Health Facts® database contains the drug name (generic and brand), national drug code (NDC), medication volume, administration times, and administration frequency that were requested and dispensed from the pharmacy. Infusion times were available, but sparsely recorded and were therefore not utilized. Medications were assumed to be administered to patients during the times the pharmacy dispensed them to be administered. The duration of medication administration was rounded to the nearest hour. If a single dose of medication was administered in an hour – the patient was considered to have received one hour of medication.

To assess medications by class (ie. IV Opioid medications), the medication table was linked to an industry standard for medication classification, Multum™ , via the NDC number.[30,31] Multum™ provides information about medications therapeutic action in three categories from general mechanism of action to a specific therapeutic category. Table A provides a summary distribution of the first IV opioid administered in the intervention and control groups before and after propensity score matching.

Table A: First IV Opioid Medication Administered in the Control and Intervention Groups Before and After Propensity Score Matching

**First IV Opioid Medication Administered**	**Before Propensity Score Matching**			**After Propensity Score Matching**		
	***Control*[1]** *n = 102,840* *n(%)*	***Intervention*[2]** *n = 1,739* *n(%)*		***Control*[1]** *n = 839* *n(%)*	***Intervention*[2]** *n = 839* *n(%)*	***p***
**Fentanyl**	41,136 (40.0)	682 (39.2)	P<0.045	337 (40.2)	338 (40.3)	P=0.432
**Morphine**	43,707 (42.5)	753 (43.3)		356 (42.4)	353 (42.1)	
**Hydromorphone**	17,380 (16.9)	301 (17.3)		144 (17.2)	146 (17.4)	
**Meperidine**	514 (0.5)	3 (0.2)		2 (0.2)	2 (0.2)	
**Nalbuphine**	103 (0.1)	0 (0.0)		0 (0.0)	0 (0.0)	

1. Control group includes patients who received IV opioids without IV acetaminophen.
2. Intervention group includes patients who received IV acetaminophen followed by IV opioids.

© 2021 Patel AK et al. *JAMA Network Open.*

eFigure 1. Forest Plots of patients in the control (IV opioids only) and intervention (IV acetaminophen followed by IV opioids) groups pre and post propensity score matching revealing a well-balanced, matched cohort of patients post propensity score matching. Odds ratios reflect a propensity for receipt of IV acetaminophen

1a. Pre-Propensity Score Matching

Baseline Characteristic	IV Opioids Only	IV Acetaminophen and IV Opioids	Odds Ratio	P-value
Circulatory	19 (2.3%)	18 (2.1%)	0.95(0.49 - 1.82)	0.8680
Digestive System	164 (19.5%)	159 (19%)	0.96(0.75 - 1.23)	0.7569
Genitourinary System	38 (4.5%)	39 (4.6%)	1.03(0.65 - 1.62)	0.9071
Musculoskeletal System	41 (4.9%)	52 (6.2%)	1.29(0.84 - 1.96)	0.2405
Dermatology System	13 (1.5%)	18 (2.1%)	1.39(0.68 - 2.86)	0.3647
Nervous System	79 (9.4%)	85 (10.1%)	1.08(0.79 - 1.5)	0.6218
Endocrine System	57 (6.8%)	47 (5.6%)	0.81(0.55 - 1.21)	0.3113
Injury and Poisonings	161 (19.2%)	175 (20.9%)	1.11(0.87 - 1.41)	0.3931
**Operations**	n=839	n=839		.
Musculoskeletal System	98 (11.7%)	91 (10.8%)	0.92(0.68 - 1.25)	0.5888
Digestive System	69 (8.2%)	74 (8.8%)	1.08(0.77 - 1.52)	0.6620
Cardiovascular System	29 (3.5%)	29 (3.5%)	1(0.59 - 1.69)	1.0000
Nervous System	22 (2.6%)	24 (2.9%)	1.09(0.61 - 1.97)	0.7649
Integumentary System	24 (2.9%)	17 (2%)	0.7(0.37 - 1.32)	0.2684
Genitourinary System	23 (2.7%)	17 (2%)	0.73(0.39 - 1.38)	0.3370
Respiratory System	22 (2.6%)	15 (1.8%)	0.68(0.35 - 1.31)	0.2445
Endocrine and Lymphatic System	10 (1.2%)	11 (1.3%)	1.1(0.47 - 2.61)	0.8262

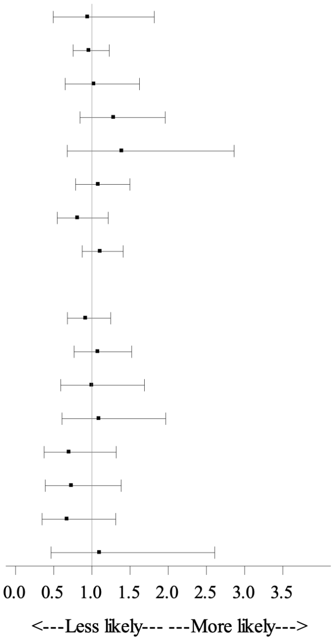

© 2021 Patel AK et al. *JAMA Network Open.*

Baseline Characteristic	IV Opioids Only	IV Acetaminophen and IV Opioids	Odds Ratio	P-value
Circulatory	2540 (2.5%)	39 (2.2%)	0.91(0.66 - 1.25)	0.5447
Digestive System	16334 (15.9%)	344 (19.8%)	1.31(1.16 - 1.47)	<.0001
Genitourinary System	3884 (3.8%)	74 (4.3%)	1.13(0.9 - 1.43)	0.2997
Musculoskeletal System	4316 (4.2%)	130 (7.5%)	1.84(1.54 - 2.21)	<.0001
Dermatology System	3258 (3.2%)	36 (2.1%)	0.65(0.46 - 0.9)	0.0093
Nervous System	6852 (6.7%)	163 (9.4%)	1.45(1.23 - 1.71)	<.0001
Endocrine System	4250 (4.1%)	98 (5.6%)	1.39(1.13 - 1.7)	0.0019
Injury and Poisonings	31193 (30.3%)	341 (19.6%)	0.56(0.5 - 0.63)	<.0001
**Operations**	n=102840	n=1739		.
Musculoskeletal System	8857 (8.6%)	205 (11.8%)	1.42(1.22 - 1.64)	<.0001
Digestive System	9508 (9.2%)	160 (9.2%)	0.99(0.84 - 1.17)	0.9491
Cardiovascular System	8734 (8.5%)	71 (4.1%)	0.46(0.36 - 0.58)	<.0001
Nervous System	5198 (5.1%)	57 (3.3%)	0.64(0.49 - 0.83)	0.0008
Integumentary System	4318 (4.2%)	41 (2.4%)	0.55(0.4 - 0.75)	0.0001
Genitourinary System	3068 (3%)	50 (2.9%)	0.96(0.73 - 1.28)	0.7928
Respiratory System	3368 (3.3%)	40 (2.3%)	0.7(0.51 - 0.95)	0.0232
Endocrine and Lymphatic System	956 (0.9%)	17 (1%)	1.05(0.65 - 1.7)	0.8363

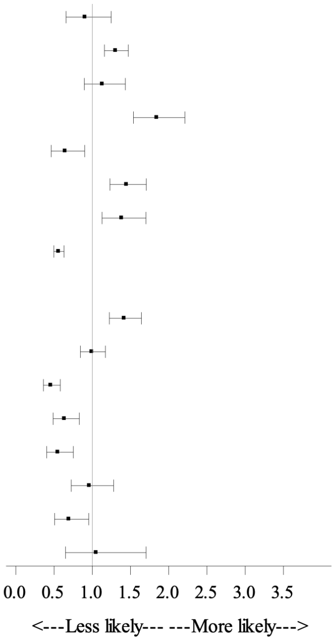

Baseline Characteristic	IV Opioids Only	IV Acetaminophen and IV Opioids	Odds Ratio	P-value
**Admin Year**	n=102840	n=1739		<.0001
2011	17651 (17.2%)	59 (3.4%)	0.1(0.08 - 0.13)	.
2012	477 (0.5%)	2 (0.1%)	0.13(0.03 - 0.51)	.
2013	28279 (27.5%)	381 (21.9%)	0.4(0.34 - 0.47)	.
2014	28217 (27.4%)	568 (32.7%)	0.6(0.52 - 0.7)	.
2015	20308 (19.7%)	465 (26.7%)	0.69(0.59 - 0.8)	.
2016	7908 (7.7%)	264 (15.2%)	Reference	.
**Census Region**	n=102840	n=1739		<.0001
Midwest	18911 (18.4%)	210 (12.1%)	1.27(1.06 - 1.52)	.
North	19776 (19.2%)	625 (35.9%)	3.61(3.13 - 4.15)	.
South	31866 (31%)	621 (35.7%)	2.22(1.93 - 2.56)	.
West	32287 (31.4%)	283 (16.3%)	Reference	.
**Bed Size Range**	n=102840	n=1739		<.0001
100-199	8539 (8.3%)	110 (6.3%)	1.55(1.23 - 1.96)	.
200-299	24604 (23.9%)	182 (10.5%)	0.89(0.73 - 1.09)	.
300-499	16692 (16.2%)	283 (16.3%)	2.04(1.7 - 2.44)	.
500+	28484 (27.7%)	960 (55.2%)	4.05(3.48 - 4.72)	.
<100	24521 (23.8%)	204 (11.7%)	Reference	.
**Teaching Facility**	n=102222	n=1737		<.0001
Yes	68515 (67%)	1489 (85.7%)	2.95(2.58 - 3.38)	.
no	33707 (33%)	248 (14.3%)	Reference	.

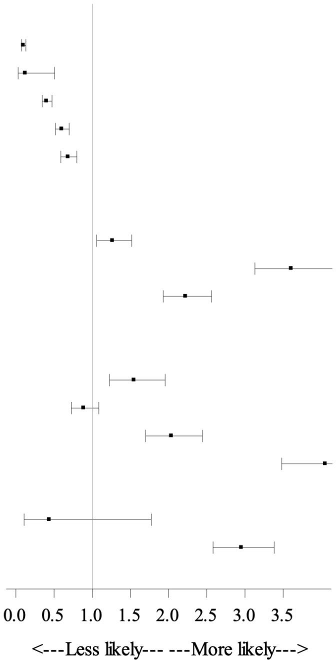

© 2021 Patel AK et al. *JAMA Network Open.*

1b. Post-Propensity Score Matching

Baseline Characteristic	IV Opioids Only	IV Acetaminophen and IV Opioids	Odds Ratio	P-value
**Age Group**	n=839	n=839		0.2750
age <2 years	102 (12.2%)	83 (9.9%)	0.71(0.49 - 1.02)	.
age ≥ 13 - 22 years	503 (60%)	493 (58.8%)	0.85(0.66 - 1.09)	.
age ≥ 2 - 6 years	82 (9.8%)	88 (10.5%)	0.93(0.64 - 1.35)	.
age ≥ 6 - 13 years	152 (18.1%)	175 (20.9%)	Reference	.
**Race**	n=839	n=839		0.9420
African American	202 (24.1%)	206 (24.6%)	1(0.74 - 1.34)	.
Caucasian	484 (57.7%)	477 (56.9%)	0.97(0.75 - 1.25)	.
Others	153 (18.2%)	156 (18.6%)	Reference	.
**Gender**	n=839	n=839		0.3280
Female	388 (46.2%)	408 (48.6%)	1.1(0.91 - 1.33)	.
Male	451 (53.8%)	431 (51.4%)	Reference	.
**Ventilation Group**	n=839	n=839		0.1190
Mechanical Ventilation	19 (2.3%)	12 (1.4%)	0.63(0.3 - 1.3)	.
Non-Invasive Mechanical Ventilation	1 (0.1%)	5 (0.6%)	4.98(0.58 - 42.74)	.
no	819 (97.6%)	822 (98%)	Reference	.
**InitialTemperature Group**	n=839	n=839		0.9280
InitialTemperature <38	768 (91.5%)	766 (91.3%)	0.91(0.56 - 1.49)	.
InitialTemperature >38.5	39 (4.6%)	38 (4.5%)	0.89(0.46 - 1.72)	.
Temperature 38 -38.5	32 (3.8%)	35 (4.2%)	Reference	.
**Diagnosis**	n=839	n=839		.
Congenital	145 (17.3%)	126 (15%)	0.85(0.65 - 1.1)	0.2075
Neoplasms	61 (7.3%)	59 (7%)	0.96(0.67 - 1.4)	0.8497

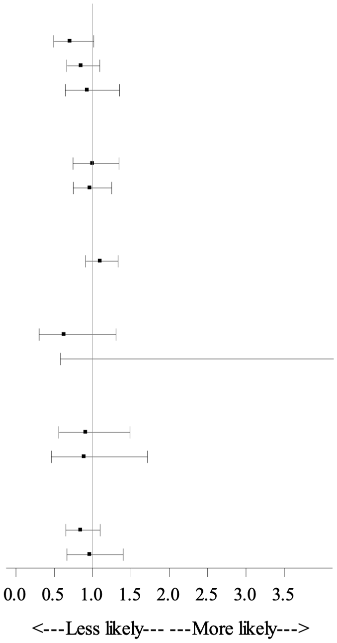

© 2021 Patel AK et al. *JAMA Network Open.*

Baseline Characteristic	IV Opioids Only	IV Acetaminophen and IV Opioids	Odds Ratio	P-value
Circulatory	19 (2.3%)	18 (2.1%)	0.95(0.49 - 1.82)	0.8680
Digestive System	164 (19.5%)	159 (19%)	0.96(0.75 - 1.23)	0.7569
Genitourinary System	38 (4.5%)	39 (4.6%)	1.03(0.65 - 1.62)	0.9071
Musculoskeletal System	41 (4.9%)	52 (6.2%)	1.29(0.84 - 1.96)	0.2405
Dermatology System	13 (1.5%)	18 (2.1%)	1.39(0.68 - 2.86)	0.3647
Nervous System	79 (9.4%)	85 (10.1%)	1.08(0.79 - 1.5)	0.6218
Endocrine System	57 (6.8%)	47 (5.6%)	0.81(0.55 - 1.21)	0.3113
Injury and Poisonings	161 (19.2%)	175 (20.9%)	1.11(0.87 - 1.41)	0.3931
**Operations**	n=839	n=839		.
Musculoskeletal System	98 (11.7%)	91 (10.8%)	0.92(0.68 - 1.25)	0.5888
Digestive System	69 (8.2%)	74 (8.8%)	1.08(0.77 - 1.52)	0.6620
Cardiovascular System	29 (3.5%)	29 (3.5%)	1(0.59 - 1.69)	1.0000
Nervous System	22 (2.6%)	24 (2.9%)	1.09(0.61 - 1.97)	0.7649
Integumentary System	24 (2.9%)	17 (2%)	0.7(0.37 - 1.32)	0.2684
Genitourinary System	23 (2.7%)	17 (2%)	0.73(0.39 - 1.38)	0.3370
Respiratory System	22 (2.6%)	15 (1.8%)	0.68(0.35 - 1.31)	0.2445
Endocrine and Lymphatic System	10 (1.2%)	11 (1.3%)	1.1(0.47 - 2.61)	0.8262

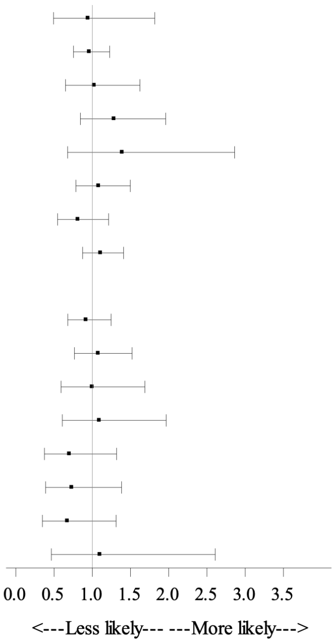

Baseline Characteristic	IV Opioids Only	IV Acetaminophen and IV Opioids	Odds Ratio	P-value
**Admin Year**	n=839	n=839		0.1770
2011	28 (3.3%)	31 (3.7%)	0.93(0.54 - 1.62)	.
2013	201 (24%)	214 (25.5%)	0.9(0.67 - 1.19)	.
2014	445 (53%)	397 (47.3%)	0.75(0.59 - 0.96)	.
2015	1 (0.1%)	2 (0.2%)	1.68(0.15 - 18.72)	.
2016	164 (19.5%)	195 (23.2%)	Reference	.
**Census Region**	n=839	n=839		0.9280
Midwest	100 (11.9%)	105 (12.5%)	1.07(0.74 - 1.53)	.
North	279 (33.3%)	268 (31.9%)	0.97(0.73 - 1.3)	.
South	319 (38%)	327 (39%)	1.04(0.79 - 1.38)	.
West	141 (16.8%)	139 (16.6%)	Reference	.
**Bed Size Range**	n=839	n=839		0.9820
100-199	52 (6.2%)	49 (5.8%)	0.88(0.55 - 1.42)	.
200-299	138 (16.4%)	136 (16.2%)	0.92(0.64 - 1.32)	.
300-499	164 (19.5%)	167 (19.9%)	0.95(0.67 - 1.35)	.
500+	387 (46.1%)	382 (45.5%)	0.92(0.68 - 1.26)	.
<100	98 (11.7%)	105 (12.5%)	Reference	.
**Teaching Facility**	n=839	n=839		0.7340
Yes	714 (85.1%)	709 (84.5%)	0.95(0.73 - 1.25)	.
no	125 (14.9%)	130 (15.5%)	Reference	.

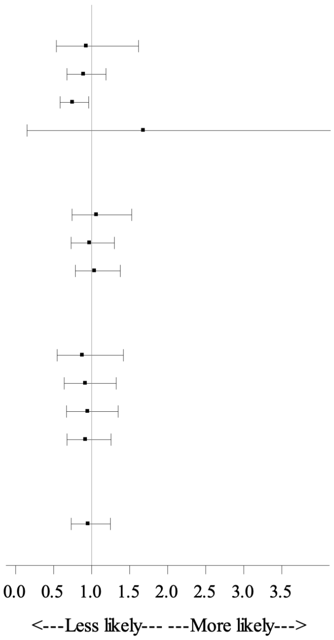

© 2021 Patel AK et al. *JAMA Network Open.*
